# Antifungal efficacy of chitosan extracted from shrimp shell on strawberry (*Fragaria* × *ananassa*) postharvest spoilage fungi

**DOI:** 10.1016/j.heliyon.2024.e29286

**Published:** 2024-04-04

**Authors:** Abir El-araby, Walid Janati, Riaz Ullah, Nisar Uddin, Ahmed Bari

**Affiliations:** a Functional Ecology and Environment Engineering Laboratory, Faculty of Science and Technology, Sidi Mohamed Ben Abdellah University, Fez, 30050, Morocco; b Department of Pharmacognosy, College of Pharmacy, King Saud University, Riyadh, 11451, Saudi Arabia; c Biofuels Institute, School of Emergency Management, School of the Environment and Safety Engineering, Jiangsu University, Zhenjiang, 212013, Jiangsu, China; d Department of Pharmaceutical Chemistry, College of Pharmacy, King Saud University, Riyadh, 11451, Saudi Arabia

**Keywords:** Chitosan, Antifungal activity, Strawberry, *Aspergillus niger*, *Botrytis cinerea*, *Fusarium oxysporum*, *Rhizopus stolonifer*

## Abstract

The strong demand for biological materials in the food industry places chitosan at the forefront of other biopolymers. The present study aims to evaluate the antifungal properties of chitosan extracted from shrimp shell waste (*Parapenaeus longirostris*) against post-harvest strawberry (*Fragaria* × *ananassa*) spoilage fungi. The physicochemical characteristics (DD, Mw, and solubility) of extracted chitosan were determined. In addition, functional characteristics were studied by Fourier transform infrared spectroscopy (FTIR), X-ray diffraction (XRD), and scanning electron microscopy (SEM). The antifungal effect of chitosan on mycelial growth and spore germination of *Aspergillus niger*, *Botrytis cinerea*, *Fusarium oxysporum*, and *Rhizopus stolonifer* was evaluated. Yield, degree of deacetylation, molecular weight, and solubility were 21.86%, 83.50%, 180 kDa, and 80.10%, respectively. A degree of deacetylation of 81.27% was calculated from the FTIR spectrum and a crystallinity index of 79.83% was determined from the X-ray diffraction pattern. SEM images of extracted chitosan showed a combination of fibrous and porous structure. At 3% chitosan, mycelial growth inhibition rates of *A. niger*, *B. cinerea*, *F. oxysporum*, and *R. stolonifer* ranged from 81.37% to 92.70%. At the same chitosan concentration, the percentages of spore germination inhibition of the isolated fungi ranged from 65.47% to 71.48%. The antifungal activity was highly dose-dependent. As a natural polymer, chitosan offers a convincing alternative to synthetic antimicrobials for the post-harvest preservation of strawberries. Its potential lies in its ability to inhibit the growth of spoilage fungi.

## 1 Introduction

Fungal contamination is a major challenge for the horticultural industry, particularly in the post-harvest handling of fruit and vegetables. Fungi are ubiquitous in the environment and can easily infect horticultural products, leading to spoilage, reduced shelf life, and economic losses. Due to their delicate nature and high water content, strawberries are one of the most perishable fruits, with a very limited storage period. Several factors contribute to their rapid degradation and decay after harvest, making proper handling and storage essential to preserve their nutritional quality. Fungal infections are often the main cause of post-harvest losses in strawberries, resulting in considerable damage. Several fungal strains commonly affect strawberries during the post-harvest period, causing rot and reducing fruit quality. Among the various fungal pathogens, some are more widespread and cause the most destructive post-harvest diseases of strawberries, such as *Botrytis cinerea* (gray rot) [1], *Rhizopus stolonifer* (soft rot) [2], and *Colletotrichum acutatum* (anthracnose) [3]. This contamination by fungal strains can occur at any stage of the post-harvest process, from harvesting to storage and transport of the fruit [4,5]. It is therefore crucial to develop effective strategies to avoid rapid decay and minimize fruit losses. Several practices have been widely applied in post-harvest, such as rapid cooling, cold storage, controlled atmosphere (CO_2_ and humidity levels), and the gamma-ray technique. Active food packaging remains a promising and innovative method capable of extending shelf life, improving safety, reducing spoilage, and maintaining the quality of packaged products, thus helping to reduce food waste [6,7].

As a natural polymer, chitosan has been considered a sustainable alternative to chemical antimicrobials and antioxidants in the food industry due to its active functional groups and excellent biological activities. Its antimicrobial properties reside primarily in the electrostatic interactions between the positively charged NH^3+^ groups of chitosan and the negatively charged cell walls [8]. This leads to a change in permeability and leakage of solutes out of the cells, preventing normal cell metabolism and subsequent cell death [9]. However, it has been indicated that the mechanism of action of the polymer may depend strongly on several factors such as the degree of deacetylation and molecular weight of chitosan [10], the type of microorganisms targeted [11], and the pH of the solvent. At lower pH values, the chitosan chain becomes almost completely protonated and, therefore, the antifungal activity will be higher [12]. Chitosan has been shown to have a positive effect on inhibiting mycelial growth and spore germination of a wide variety of food spoilage microorganisms [13,14]. Due to its unique structure and exceptional antimicrobial properties, chitosan is one of the most widely used biopolymers as natural preservatives and additives in the food sector in order to improve the storage and preservation of food products [15,16]. This promising polymer has gained significant interest due to its biodegradability, non-toxicity, and antimicrobial activity to minimize the massive and uncontrolled use of food packaging derived from petroleum-based plastics that have a negative impact on the environment [17]. Over the last few decades, chitosan has received considerable attention in food science as a reference material for the development of edible films and coatings capable of protecting foodstuffs and extending their shelf life.

The aim of the present study was to determine the physicochemical and functional characteristics of chitosan extracted from shrimp (*Parapenaeus longirostris*) shell waste. Secondly, to evaluate its *in-vitro* fungicidal potential, in terms of mycelial growth and spore germination, against common post-harvest spoilage fungi isolated from strawberries (*Fragaria* × *ananassa*).

## 2 Materials and methods

### 2.1 Extraction of chitosan from *Parapenaeus longirostris*

Chitosan extraction was performed from shrimp (*Parapenaeus longirostris*) shell waste and according to the process described by Ref. [18]. First, the shells were washed, dried at 100 °C, and powdered. After that, the shell powder was deproteinized with NaOH (1.25 N) in a ratio of 1:8 (w/v) at 70 °C for 3 h. The resulting product was recovered, washed, and dried at 80 °C. Then, the demineralization step was performed with an organic acid, citric acid, at 4% in a ratio of 1:10 (w/v) for 24 h at room temperature and under constant stirring (250 rpm). The resulting chitin was collected, washed, and dried at 80 °C. The last step was the conversion of chitin to chitosan by alkaline deacetylation. This step was performed with NaOH (12.5 N) in a ratio of 1:5 (w/v) at 100 °C for 12 h. The resulting chitosan was filtered, washed, and dried at 80 °C. Extraction yield (%) was determined.

### 2.2 Physicochemical characteristics of extracted chitosan

#### 2.2.1 Degree of deacetylation (DD)

The DD of the extracted chitosan was determined by the acid-base titration method [19]. The chitosan sample was dissolved in HCl solution (0.1 M) at room temperature under constant stirring. Then, 2–3 drops of 1% methyl orange indicator were added. The colored solution was titrated with NaOH solution (0.1 M) until a yellow color appeared. The DD (%) was calculated according to the following formula (Equation (1)):

(1) $$DD\left(\%\right)=\frac{\left(C_{1}\times V_{1}\;-\;C_{2}\times V_{2}\right)}{G\times \left(100\;-\;W\right)\times 0.0994}\times 0.016$$

• **C**_**1**_ and **C**_**2**_**:** Concentrations of HCl and NaOH solution, respectively;
• **V**_**1**_ and **V**_**2**_**:** Volumes of HCl and NaOH solution, respectively;
• **0.016:** Equivalent weight of NH_2_ groups corresponding to 1 mL of HCl;
• **G:** Weight of extracted chitosan;
• **W:** Percentage of water in chitosan;
• **0.0994:** Theoretical equivalent mass of NH_2_ groups.

#### 2.2.2 Molecular weight (Mw)

A rheological study was performed to determine the intrinsic viscosity [η] of the extracted chitosan and thus the Mw, using an Ubbelohde capillary viscometer at 25 °C. Different solutions of chitosan were prepared (0.1%, 0.05%, 0.025%, and 0.0125%) and dissolved in 0.3 M HAc/0.2 M NaAc buffer [20]. The Mw of chitosan extracted with citric acid was determined by the Mark-Houwink-Sakurada formula (Equation (2)). The empirical constants were determined (K = 9.55*10^−2^ and α = 0.73) with reference to previous work [21]. K and α depend mainly on the polymer nature, the solvent characteristics, and the reaction temperature.

(2) [η]=K.Mwα

#### 2.2.3 Solubility

Solubility was determined by dissolving the chitosan sample in a 1% acetic acid solution at 60 °C with constant stirring for 24 h. The solution was then subjected to centrifugation (4000 rpm). The pellet (undissolved solid) was dried at 60 °C overnight. The solubility of the extracted chitosan was calculated according to the following formula (Equation (3)):

(3) $$Solubility\;\left(\%\right)=\frac{\left(W_{I+S}\;-\;W_{F+S}\right)}{\left(W_{I+S}\;-\;W_{I}\right)}\times 100$$

• **W**_**I**_**:** Initial weight of tube;
• **W**_**I + S**_ and **W**_**F + S**_**:** Initial and the final weight of tube + sample, respectively.

### 2.3 Functional characterization of extracted chitosan

The shrimp shell chitosan was characterized by Fourier transform infrared spectroscopy, X-ray diffraction, and scanning electron microscopy. This characterization is a crucial step in understanding the material's properties and behavior, enabling the optimization of chitosan-based materials for various applications in fields such as biotechnology, medicine, and food science.

#### 2.3.1 Fourier transform infrared (FTIR) spectroscopy

The extracted chitosan was characterized by FTIR spectroscopy to determine its functional groups and molecular structure. The FTIR spectrum was obtained in a frequency range from 400 to 4000 cm^−1^ using a spectrometer (Bruker Vertex 70). The degree of deacetylation of chitosan was estimated from the intensity ratio between the peak at 1655 cm^−1^ and that at 3450 cm^−1^ [22].

#### 2.3.2 X-ray diffraction (XRD)

X-ray diffraction was used to characterize the crystalline structure of extracted chitosan by measuring the intensity of diffraction peaks at characteristic angles. The diffraction pattern was obtained over a 2θ scan range from 5 to 90° using a diffractometer (X'Pert-Pro). The crystallinity index of chitosan was calculated by comparing the area under crystalline peaks to the total area under all peaks (crystalline and amorphous) [23].

#### 2.3.3 Scanning electron microscopy (SEM)

Scanning electron microscopy was used to characterize the morphology and structure of the chitosan surface at high resolution. SEM images were obtained at an accelerating voltage of 12 Kv using a scanning electron microscope (JEOL JSM-IT500HR). The chitosan sample was coated with a thin layer of gold to improve conductivity and reduce charging effects.

### 2.4 Antifungal activity of chitosan on strawberry spoilage fungi

#### 2.4.1 Growth kinetics of isolated strains

The antifungal activity of the chitosan extracted with citric acid was evaluated against the mycelial growth of *Aspergillus niger*, *Botrytis cinerea*, *Fusarium oxysporum,* and *Rhizopus stolonifer*. Fungal strains were isolated from spoiled strawberries (*Fragaria × ananassa*) in our previous study [24]. The effect of extracted chitosan on mycelial growth of strawberry spoilage fungi was evaluated on potato dextrose agar (PDA) solid medium [25]. Chitosan samples at concentrations of 0.5, 1, 1.5, 2, 2.5, and 3% were prepared in 1% acetic acid. Mycelial discs (5 mm in diameter) were aseptically cut from the periphery of viable cultures (one-week-old) of each fungus and grown on PDA plates that had been amended by incorporating increasing concentrations of extracted chitosan (from 0.5 to 3%). This experiment was repeated twice over time with three replicates. Afterwards, the Petri plates were incubated for 7 days in darkness at 25 °C. The growth kinetics of molds isolated from spoiled strawberries were determined by measuring mycelial growth diameters. The following formula (Equation (4)) was used to calculate fungal growth rates (μ):

(4) $$\mu =\frac{\left[{lnD}_{2}\;-\;{lnD}_{1}\right]\;}{\Delta t}$$

• **μ:** Fungal growth rate (μ on day^−1^);
• **D**_**2**_ and **D**_**1**_**:** Mycelial growth diameters;
• **Δt:** Time Unit (t_2_-t_1_).

#### 2.4.2 Effect of chitosan on mycelial growth

After 7 days of incubation, the inhibition rate (%) of mycelial growth of *A. niger*, *B. cinerea*, *F. oxysporum*, and *R. stolonifer* on PDA plates was determined using the radial growth of mycelium in the absence of chitosan (negative control) and in the presence of increasing concentrations of extracted chitosan (from 0.5 to 3%) according to the following formula (Equation (5)):

(5) $$Inhibition\;\left(\%\right)=\frac{\left[R_{1}\;-\;R_{2}\right]}{R_{1}}\times 100$$

• **R**_**1**_: Radial growth of mycelium in the absence of chitosan;
• **R**_**2**_**:** Radial growth of mycelium in the presence of chitosan.

#### 2.4.3 Effect of chitosan on spore germination

Spores were collected from 10-day-old fungal colony cultures of each isolated fungi and suspended with sterile distilled water containing 0.1% (v/v) Tween 80 [25]. The spore suspensions were filtered with Whatman paper (No.1) to separate the spores from the mycelium and debris of the solid PDA medium, and then the spore concentrations were adjusted with sterile distilled water to 10^4^ spores mL^−1^. To evaluate the effect of chitosan on spore germination, prepared spore suspensions of *A. niger*, *B. cinerea*, *F. oxysporum*, and *R. stolonifer* were added to liquid potato dextrose broth (v/v) amended with increasing concentrations of extracted chitosan (from 0.5 to 3%). Cultures were incubated for 24 h at 25 °C. This experiment was repeated twice over time with three replicates. After the incubation period, the germination of 100 spores per plates was assessed under a microscope using a micrometer. A spore was considered germinated when the germ tube length was equal to or longer than the spore length [25]. The inhibition rate (%) of spore germination of isolated fungus was determined using the following formula (Equation (6)):

(6) $$Inhibition\;\left(\%\right)=\frac{\left[N_{1}\;-\;N_{2}\right]}{N_{1}}\times 100$$

• **N**_**1**_: Average number of spores germinated in the absence of chitosan;
• **N**_**2**_**:** Average number of spores germinated in the presence of chitosan.

### 2.5 Statistical analyses

Statistical analyses were performed using SPSS statistical software. All data were analyzed for significant differences by applying the analysis of variance (ANOVA) test, with p ≤ 0.05. The obtained *in-vitro* datasets were presented as mean ± standard deviation. The mathematical model of the growth rate of the isolated strains was established using Microsoft Excel 2013.

## 3 Results and discussion

### 3.1 Physicochemical characteristics of extracted chitosan

Chitosan was extracted using an organic acid during the demineralization step of shrimp (*Parapenaeus longirostris*) shell. The use of citric acid for chitosan extraction avoids the use of mineral acids, which is more environmentally friendly and promising for industrial food applications. The yield, degree of deacetylation (DD), molecular weight (Mw), and solubility of the extracted chitosan were determined (Table 1).

**Table 1:** Physicochemical characteristics of shrimp chitosan extracted with citric acid.

	Yield (%)	DD (%)		Mw (kDa)	Solubility (%)
		Titration	FTIR		
Shrimp shell chitosan	21.86	83.50	81.27	180	80.10

Indeed, the physicochemical characteristics of chitosan can vary significantly depending on the source material and the extraction process used. Optimizing these conditions is crucial to obtaining the desired characteristics for specific applications. Chitosan chemically extracted from the shell of shrimp (*Litopenaeus vannamei*) was shown to have a deacetylation degree of 65% [26]. The DD values determined by the acid-base titration method of chitosan extracted from shrimp (*Penaeus notialis*) and crab (*Callinectes amnicola*) shells were 89.73% and 84.20%, respectively. Chitosan extracted from soil fungi (*Cunninghamella echinulata*) showed a deacetylation degree of 80.88% [27]. The shrimp shell produced a higher chitosan yield (16.93%) than the crab shell (13.29%) [28]. It has been reported that the extraction process affects the yield of chitosan extracted from the carapace of mantis shrimp (*Oratosquilla nepa*). The yield increased from 14.13% to 15.79% when the deacetylation time of chitin was increased from 2 to 4 h [29]. Extraction conditions and polymer initial source are crucial in determining the yield of chitosan [30]. Öğretmen et al. [31] extracted chitosan from the shell waste of pink shrimp (*Parapenaeus longirostris*). Physicochemical characterization showed that the degree of deacetylation, solubility, and molecular weight were 81.50%, 86.79%, and 310 kDa, respectively. Deacetylation time can significantly influence the molecular weight of chitosan extracted from the carapace of deep-sea mud shrimp (*Solenocera hextii*). The molecular weights were 263.95, 52.61, and 5.15 kDa for 1.5, 3, and 6 h, respectively [32]. Demineralization and deproteinization by lactic acid bacteria and deacetylation by deacetylases produced a shrimp chitosan with a deacetylation degree of 78%, a solubility of 25%, and a molecular weight of 71.31 KDa [33]. Previous studies have revealed that the deacetylation degree and the molecular weight are vital parameters of chitosan that significantly control its solubility and pH sensitivity, thus affecting its biological and functional properties [34,35]. These physicochemical characteristics are crucial to the use of chitosan in various applications, as they determine its suitability for specific uses and processing conditions.

### 3.2 Functional characterization of extracted chitosan

#### 3.2.1 Fourier transform infrared (FTIR) spectroscopy

**Fig. 1:**
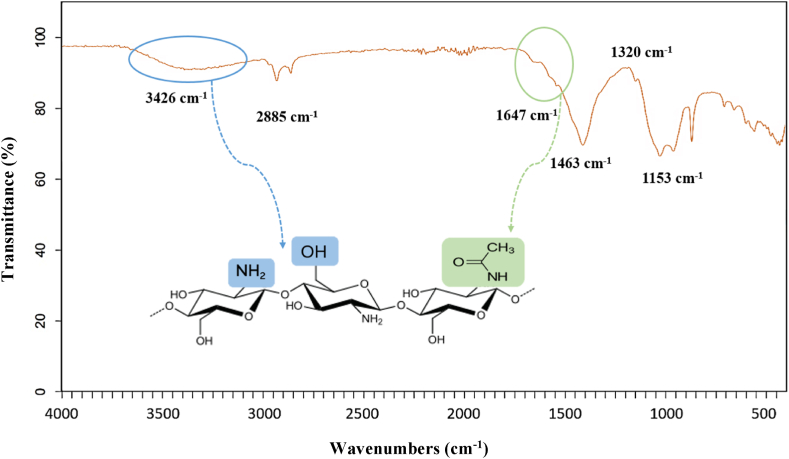
FTIR spectrum of shrimp shell chitosan extracted with citric acid.

Chitosan extracted with citric acid was characterized by Fourier transform infrared spectroscopy. The interpretation of the FTIR spectrum (Fig. 1) involves comparing the peaks observed with reference spectra from the literature [36,37] and understanding the vibrations associated with specific functional groups.

IR spectral data for chitosan revealed characteristic absorption bands at 3426 cm^−1^ (N–H and O–H stretching vibrations), 2885 cm^−1^ (CH_2_, CH_3_ aliphatic stretching vibration), 1647 cm^−1^ (NH bending vibration), 1463 cm^−1^ (CH_3_ symmetrical deformation), 1320 cm^−1^ (Amide III band vibration), and 1153 cm^−1^ (C–*O*–C bridge stretching). The FTIR spectrum can also show bands linked to other functional groups or impurities, enabling chitosan samples to be identified and characterized. The degree of deacetylation was determined to be 81.27%.

#### 3.2.2 X-ray diffraction (XRD)

**Fig. 2:**
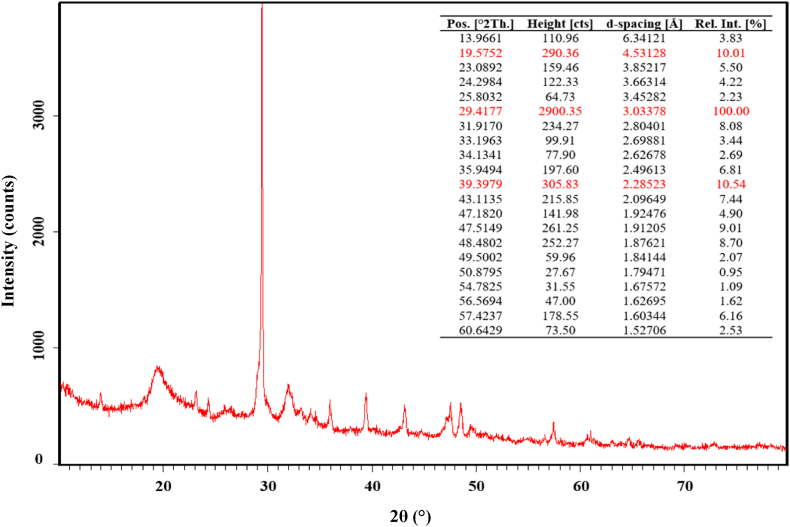
X‐ray diffraction pattern of shrimp shell chitosan extracted with citric acid.

Chitosan extracted with an organic acid was characterized by X-ray diffraction (Fig. 2) and the crystallinity index was determined to be 79.83%. The diffraction pattern shows two sharper peaks at 2θ around 20° (290.36 counts/s) and 40° (305.83 counts/s) and a maximum diffraction peak around 30° (2900.35 counts/s). Similar results have been obtained in previous studies.

The XRD pattern of chitosan, extracted from the shell of chiton (a mollusc), revealed the strongest and sharpest diffraction reflection at 2θ around 30–35° (625 counts/s) [38]. Another study reported that chitosan extracted from horse mussel exhibited a maximum diffraction peak at 2θ around 20.04° (113.92 counts/s) [39]. According to the literature, the crystalline structure of chitin can vary from one source to another, leading to differences in the structure of the chitosan obtained.

#### 3.2.3 Scanning electron microscopy (SEM)

The morphological characteristics of chitosan extracted with citric acid were studied by scanning electron microscopy. The chitosan material has been gold-coated to ensure clear, precise images.

**Fig. 3:**
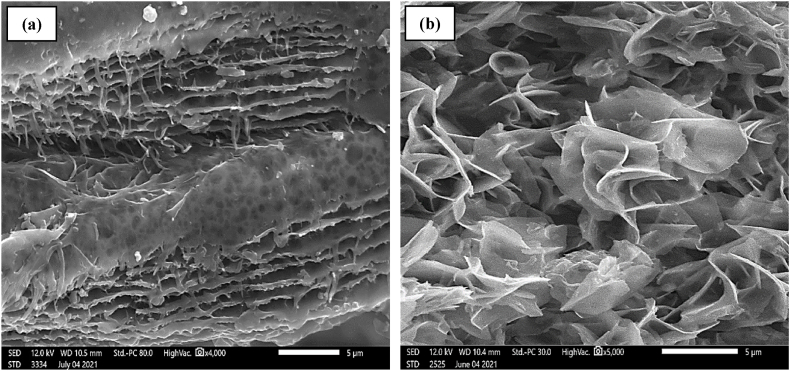
SEM images of shrimp shell chitosan extracted with citric acid.

SEM images show a combination of fibrous and porous structure. Fig. 3 (a) illustrates the arrangement and alignment of chitosan fibers, while Fig. 3 (b) reveals porosity and pore distribution. The presence of fibers and pores in the extracted polymer is in line with previous studies on chitosan from different sources and with different physicochemical characteristics.

### 3.3 Antifungal activity of chitosan on strawberry spoilage fungi

#### 3.3.1 Growth kinetics of isolated strains

**Fig. 4:**
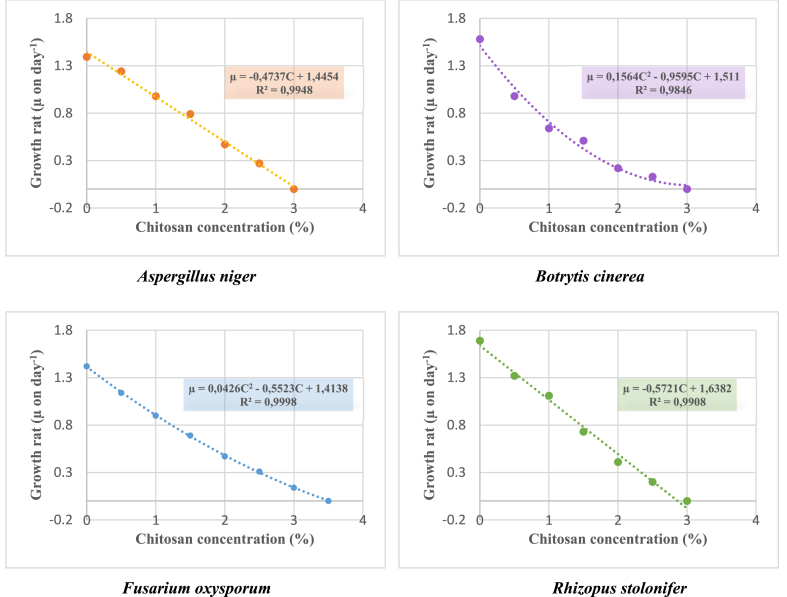
Fungal growth rates (μ) of strawberry spoilage fungi according to increasing chitosan concentrations (C). R^2^: Coefficient of determination.

The growth kinetics of *A. niger*, *B. cinerea*, *F. oxysporum*, and *R. stolonifer* isolated from spoiled strawberries (*Fragaria* × *ananassa*) were determined by measuring mycelial growth diameters during 7 days. The following figure (Fig. 1) shows the fungal growth rates (μ on day^−1^) of strawberry spoilage fungi as a function of increasing concentrations of chitosan extracted with citric acid (from 0.5 to 3%). Chitosan revealed strong dose-dependent (0.5–3%) antifungal activity on the growth of all strains isolated from spoiled strawberries. Fig. 4 shows that the rate of fungal growth decreased significantly with increasing chitosan concentration. At a concentration of 3% chitosan, almost complete inhibition of the mycelial growth of the tested strains was observed. It is useful to work on microcosms (Petri dishes) before returning to the evaluation of the fungal growth on strawberry fruits (*in-vivo*). Extracted chitosan showed a negative effect on fungal mold growth, demonstrating its ability to reduce significantly the microbial load of strawberry fruit spoilage. The effect of chitosan on fungal growth rates is translated into two models: a linear model reflecting a specific resistance of *A. niger* (μ = −0.4737C + 1.4454) and *R. stolonifer* (μ = −0.5721C + 1.6382) and a polynomial model representing a sensitivity of *B. cinerea* (μ = 0.1564C^2^ – 0.9595C + 1.511) and *F. oxysporum* (μ = 0.0426C^2^ – 0.5523C + 1.4138) in response to chitosan as a natural antifungal material. This difference might be attributed to structural differences in the walls of the isolated fungi. The mathematical model of the growth rate of the isolated strains can be explained by the Monod growth rate model in the absence of chitosan [40] and the Haldane growth kinetics model in the presence of chitosan as an inhibitor [41]. Studies are underway to further detail these mathematical models.

#### 3.3.2 Effect of chitosan on mycelial growth

The inhibition rate (%) of mycelial growth of *A. niger*, *B. cinerea*, *F. oxysporum*, and *R. stolonifer* on PDA plates was determined after 7 days of incubation by measuring the mycelial growth in the absence of chitosan and in the presence of increasing concentrations of extracted chitosan (from 0.5 to 3%). The following table (Table 2) shows the inhibition rates (%) of mycelial growth for all fungi evaluated. A chitosan concentration of 0.5% inhibited the mycelial growth of gray mold (*B. cinerea*) with an inhibition rate of 19.09%. At 1% chitosan, the inhibition rates of the isolated strains ranged from 23.08% to 27.83%, with the highest inhibition rate for mycelial growth of *A. niger*. The 1.5% chitosan concentration inhibited more than 50% of the mycelial growth of the fungi evaluated, with a higher inhibition rate of 63.84% for the mycelial growth of *B. cinerea*. The 2% chitosan concentration showed high rates of mycelial growth inhibition for *F. oxysporum* and *R. stolonifer* (soft rot) with 72.65% and 70.12%, respectively. At 2.5% chitosan, the high rates of mycelial growth inhibition were observed for *A. niger* and *B. cinerea* with 85.93% and 80.74%, respectively. At the highest concentration of chitosan (3%), the mycelial growth inhibition rates were 92.70%, 86.01%, 84.94%, and 81.37% for *A. niger*, *B. cinerea*, *F. oxysporum*, and *R. stolonifer*, respectively. Extracted chitosan significantly reduced the mycelial growth of strawberry spoilage fungi (P < 0.05) compared with the negative control (absence of chitosan). The higher the concentration of chitosan, the greater the percentage of inhibition potency.

**Table 2:** Inhibition rate (%) of mycelial growth of strawberry spoilage fungi under chitosan treatment after 7 days of incubation.

Chitosan concentrations	Inhibition rate (%)			
	A. niger	B. cinerea	F. oxysporum	R. stolonifer
0.5 %	15.34 ± 0.95^a^	19.09 ± 0.37^d^	17.50 ± 0.19^c^	16.72 ± 0.62^b^
1 %	27.83 ± 0.17^d^	24.76 ± 0.29^b^	23.08 ± 0.53^a^	25.96 ± 0.80^c^
1.5 %	56.72 ± 0.63^b^	63.84 ± 1.06^c^	51.26 ± 0.94^a^	50.71 ± 0.43^a^
2 %	66.98 ± 0.70^a^	69.86 ± 0.81^b^	72.65 ± 0.44^d^	70.12 ± 0.27^c^
2.5 %	85.93 ± 0.15^c^	80.74 ± 0.38^b^	79.12 ± 1.26^a^	78.24 ± 0.60^a^
3 %	92.70 ± 0.48^d^	86.01 ± 0.17^c^	84.94 ± 0.23^b^	81.37 ± 0.91^a^

The values followed by the same letter a–d in the same line show no statistically significant differences according to the Duncan test (p < 0.05). Mean values (n = 6) ± standard deviation (SD).

**Fig. 5:**
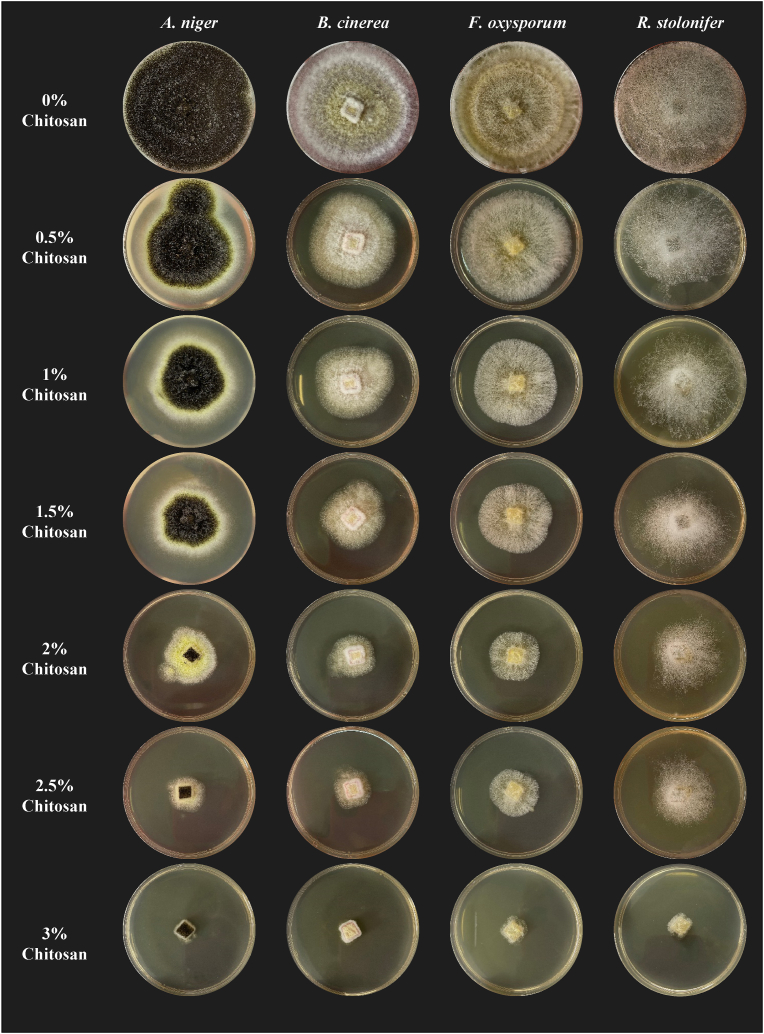
Mycelial growth of *Aspergillus niger*, *Botrytis cinerea*, *Fusarium oxysporum*, and *Rhizopus stolonifer* on PDA plates after 7 days of incubation according to increasing chitosan concentrations.

The following figure (Fig. 5) illustrates the mycelial growth of *A. niger*, *B. cinerea*, *F. oxysporum*, and *R. stolonifer* on PDA plates after 7 days of incubation as a function of increasing concentrations of chitosan extracted (0.5, 1, 1.5, 2, 2.5, and 3%). In general, the higher the concentration of chitosan, the lower the mycelial growth of the molds tested.

#### 3.3.3 Effect of chitosan on spore germination

The inhibition rate (%) of spore germination of *A. niger*, *B. cinerea*, *F. oxysporum*, and *R. stolonifer* was determined after 24 h of incubation by measuring the number of spores germinated in the absence of chitosan and in the presence of increasing concentrations of extracted chitosan (from 0.5 to 3%). Germination of 100 spores per Petri plates was assessed. The following table (Table 3) shows the inhibition rates (%) of spore germination for all fungi evaluated. The 0.5% chitosan concentration showed high spore germination inhibition rates for *B. cinerea* and *R. stolonifer* with 14.26% and 11.04%, respectively. A chitosan concentration of 1% inhibited the spore germination of the gray mold with an inhibition rate of 30.28%. At 1.5% chitosan, high inhibition rates of spore germination were observed for *F. oxysporum* and *B. cinerea* with 46.35% and 40.75%, respectively. The concentration of 2% chitosan inhibited more than 50% of the spore germination of the evaluated fungi, with a higher inhibition rate of 57.92% for *B. cinerea*. At 2.5% chitosan, the highest inhibition rates of spore germination were observed for *A. niger* and *F. oxysporum* with 64.37% and 62.84%, respectively. At the highest concentration of chitosan (3%), the inhibition rates of spore germination were 71.14%, 71.48%, 65.47%, and 68.65% for *A. niger*, *B. cinerea*, *F. oxysporum*, and *R. stolonifer*, respectively. Extracted chitosan significantly reduced spore germination rates of strawberry spoilage fungi (P < 0.05) compared with the negative control (absence of chitosan). The higher the concentration of chitosan, the lower the spore germination rates of the isolated strains.

**Table 3:** Inhibition rate (%) of spore germination of strawberry spoilage fungi under chitosan treatment after 24 h of incubation.

Chitosan concentrations	Inhibition rate (%)			
	A. niger	B. cinerea	F. oxysporum	R. stolonifer
0.5 %	6.39 ± 0.37^a^	14.26 ± 0.17^d^	8.93 ± 0.82^b^	11.04 ± 0.43^c^
1 %	22.08 ± 1.07^b^	30.28 ± 0.68^d^	27.42 ± 0.25^c^	20.55 ± 0.16^a^
1.5 %	37.96 ± 0.25^a^	40.75 ± 0.51^b^	46.35 ± 1.06^c^	38.11 ± 0.78^a^
2 %	53.70 ± 0.48^b^	57.92 ± 0.76^c^	51.93 ± 0.10^a^	53.02 ± 1.00^b^
2.5 %	64.37 ± 0.23^c^	59.74 ± 0.62^a^	62.84 ± 0.77^b^	60.43 ± 0.45^a^
3 %	71.14 ± 0.55^c^	71.48 ± 0.90^c^	65.47 ± 1.13^a^	68.65 ± 0.23^b^

The values followed by the same letter a–d in the same line show no statistically significant differences according to the Duncan test (p < 0.05). Mean values (n = 6) ± standard deviation (SD).

Chitosan has significant antifungal properties, making it a valuable compound for a variety of applications, including agriculture, food preservation, and pharmaceuticals. Chitosan's antifungal activity is attributed to its unique physicochemical characteristics, which enable it to interact with fungal cells and disrupt their normal functions. It is well-documented that chitosan is capable of inhibiting the growth of a wide variety of foodborne pathogens [42,43]. The development of chitosan-based films and coatings with numerous antifungal properties is generating growing interest and demand, with the aim of reducing the use of chemical preservatives and improving food storage and preservation [44]. In general, the antifungal activity of chitosan is affected by many factors such as the molecular weight (Mw) of the polymer, the degree of deacetylation (DD), the concentration of chitosan, and the pH of the solvent. Previous studies have revealed that differences in DD and Mw of chitosan can be directly related to the extraction process, as well as the initial raw material [45]. This antifungal activity also depends on the type of fungus targeted [46,47]. Strawberries contain high levels of bioactive compounds such as vitamins and antioxidants, but unfortunately remain highly perishable fruits that spoil quickly after harvest [16]. They are susceptible to severe post-harvest losses and therefore have a very limited shelf life. The main causes of post-harvest decay of horticultural products during storage are the development of rots caused by a range of fungi leading to severe economic losses [48]. The most common fungal pathogens affecting strawberries are *Botrytis cinerea* (gray mold), *Rhizopus stolonifer* (soft rot), *Penicillium* spp, *Aspergillus* spp, *Colletotrichum* spp. (anthracnose), and *Fusarium oxysporum* [[49], [50], [51]]. The use of bioactive compounds such as chitosan for post-harvest treatments of strawberries has received increased attention in recent years to avoid future problems with chemical fungicides. The ability of chitosan to inhibit fungal growth and improve food safety explains the interest in developing bioactive food packaging based on chitosan and its derivatives capable of extending the shelf life of strawberry fruits [52].

Various studies have shown that chitosan has broad-spectrum antifungal activity. A study by De Bona et al. [53] reported that chitosan with a degree of acetylation of 17% and a molecular weight of 173 kDa affected the growth development of *Botrytis cinerea*. Chitosan concentrations of 2 and 3 g/L provided a 40% reduction in mycelium growth compared to controls, while with concentrations of 0.5 and 1 g/L, the average reduction was 22%. Increasing concentrations of chitosan clearly showed an inhibitory effect of the polymer on the mycelial growth of *Botrytis cinerea*. Chitosan was able to inhibit significantly the growth and spore germination of *Fusarium oxysporum*. The results showed that chitosan had a dose-dependent inhibitory effect when the concentration of chitosan was below 0.4 g/L. The germination of *Fusarium oxysporum* spores was also inhibited under the chitosan concentration of 0.215 g/L [54]. A study by Mejdoub-Trabelsi et al. [55] reported that chitosan (DD of 75%–85% and Mw of 150 kDa) applied at a concentration of 4.0 g/L significantly inhibited mycelial growth of *Fusarium sambucinum*, *Fusarium oxysporum*, and *Fusarium graminearum* by 89.0%, 88.4%, and 89.8%, respectively. Low molecular weight chitosan (3.4–51.3 kDa) showed a great inhibitory effect than high molecular weight chitosan (136.8–342.0 kDa) on spore germination and mycelial growth of *Botrytis cinerea* [56]. A study by Coutinho et al. [57] reported that the chitosan samples (Mw of 132, 228, and 245 kDa and DA of 5.9, 6.8, and 6.3%) were capable of inhibiting the growth of *Penicillium citrinum* and *Penicillium mallochii*, and the inhibitory effect of the polymer was shown to be a concentration-dependent mode. Mycelial growth of *Phytophthora infestans* was completely inhibited by chitosan (DD of 95% and Mw of 100 kDa) at the concentration of 0.2 g/L. In the same report, the authors indicated that the germination rate of *Phytophthora infestans* spores was inhibited from 64.10% to 1.29% after treatment with a chitosan concentration of 0.05 g/L [58]. Another research studied the effect of chitosan (DDA of 93% and Mw of 100 kDa) on spore germination and mycelial growth of *Aspergillus ochraceus*. The results showed that the rate of spore germination was inhibited to 19% and 36% after treatment with chitosan concentrations of 0.05% and 0.1%, respectively. After 7 days of incubation, the inhibition rate of mycelial growth of *Aspergillus ochraceus* on PDA supplemented with 0.05% and 0.1% chitosan was 13.6% and 34.5%, respectively [59]. Because of its unique character, chitosan can be combined with other natural antimicrobial agents or substances to enhance its antifungal or antibacterial properties. For example, chitosan revealed a negative effect on mycelial growth, spore germination, and sporulation of *Rhizopus stolonifer* with a 48% inhibition of mycelial growth at a concentration of 2.5%. However, the combination of chitosan with sodium benzoate completely inhibited mycelial growth of the strain tested [60]. A study carried out on the post-harvest preservation of strawberries showed that conjugating chitosan with *Silene uniflora* extract enhanced its antifungal activity against two strawberry pathogens, *Botrytis cinerea* and *Colletotrichum nymphaeae* [61].

The mechanism of action of chitosan's antifungal activity is multifaceted, involving several processes that may vary according to fungal species and environmental conditions. According to the literature, the hypothesis predicts that negatively charged phospholipids in the cell membrane of the fungus can interact with the positively charged chitosan, leading to the destruction of the membrane and the entry of the polymer into the cytoplasm of the cell [43]. It appears that the positively charged amino group (-NH_2_) of chitosan is the key factor affecting its antifungal activity which is primarily considered fungistatic, rather than fungicidal [62]. The application of this promising and versatile biopolymer for the post-harvest preservation of strawberries is widely confirmed in the literature, as it inhibits the growth of spoilage pathogens, maintains freshness and quality, and thus extends the fruit's shelf life [63,64]. Due to its unique antifungal and antibacterial properties against a wide variety of food-borne pathogens, chitosan has received considerable attention in recent decades as the reference biodegradable polymer for the development of active food packaging materials to replace synthetic antimicrobials in the food industry. Researchers continue to explore and optimize the use of chitosan and its derivatives to control fungal infections and improve crop yields, food safety, and other applications.

## 4 Conclusion

Shrimp shell chitosan extracted with citric acid showed significant physicochemical characteristics: a degree of deacetylation of 83.50% and a molecular weight of 180 kDa. The extracted polymer revealed a strong antifungal effect of post-harvest strawberry spoilage fungi, notably *A. niger*, *B. cinerea*, *F. oxysporum,* and *R. stolonifer*. At the highest chitosan concentration (3%), inhibition rates for mycelial growth and spore germination reached 92.70% and 71.48%, respectively. The antifungal activity was highly dose-dependent. The results confirm that this exceptional biopolymer could be a promising alternative to synthetic antimicrobials and antioxidants for post-harvest preservation of strawberries by preventing the growth of spoilage fungi and increased oxidation. As the industry continues to prioritize sustainability, chitosan offers innovative solutions that meet both environmental and functional needs, suggesting potential applications in food preservation and packaging, and consequently in extending the shelf life of foodstuffs.

## Funding

This research received no external funding.

## Data availability statement

The original contributions presented in the study are included in the article/supplementary material, further inquiries can be directed to the corresponding author.

## CRediT authorship contribution statement

**Abir El-araby:** Writing – review & editing, Writing – original draft, Supervision, Resources, Methodology, Investigation, Conceptualization. **Walid Janati:** Writing – review & editing, Writing – original draft, Project administration, Methodology, Conceptualization. **Riaz Ullah:** Writing – review & editing, Visualization, Resources, Methodology. **Nisar Uddin:** Investigation, Formal analysis. **Ahmed Bari:** Project administration, Formal analysis.

## Declaration of competing interest

The authors declare that they have no known competing financial interests or personal relationships that could have appeared to influence the work reported in this paper.
